# Feasibility of a Type 2 Diabetes Prevention Program at Nationwide Level in General Practice: A Pilot Study in Italy

**DOI:** 10.3390/jcm13041127

**Published:** 2024-02-16

**Authors:** Rosalba La Grotta, Valeria Pellegrini, Francesco Prattichizzo, Oriana Amata, Lorenzo Panella, Antonio Frizziero, Marco Visconti, Gabriella Averame, Pier Claudio Brasesco, Ilaria Calabrese, Olga Vaccaro, Antonio Ceriello

**Affiliations:** 1 Istituto di Ricovero e Cura a Carattere Scientifico (IRCCS) MultiMedica, Via Fantoli 16/15, 20138 Milan, Italy; rosalba.lagrotta@multimedica.it (R.L.G.); valeria.pellegrini@multimedica.it (V.P.); 2 Department of Rehabilitation, Azienda Socio Sanitaria Territoriale (ASST) Gaetano Pini-Centro Specialistico Ortopedico Traumatologico (CTO), Piazza Cardinal Ferrari 1, 20122 Milan, Italy; 3 Department of Biomedical, Surgical and Dental Sciences, Università degli Studi di Milano, 20122 Milan, Italy; 4 Consorzio Sanità (Co.S.), Via Marconi 3, 26015 Soresina, Italy; 5 Medicoopliguria, Via Peschiera 33, 16121 Genova, Italy; 6 Department of Clinical Medicine and Surgery, “Federico II” University of Naples, Via Sergio Pansini, 5, 80131 Naples, Italy; ilariacalabrese@live.it (I.C.);

**Keywords:** type 2 diabetes, screening, prediabetes, prevention, physical activity, Findrisc, PAR-Q

## Abstract

Background: Lifestyle interventions halt the progression of prediabetes to frank type 2 diabetes (T2D). However, the feasibility of a diabetes prevention program promoting tailored interventions on a national scale and conducted by primary care physicians is unclear. Methods: General practitioners located in ten different regions throughout Italy enrolled random subjects without known metabolic diseases to identify individuals with prediabetes and prescribe them an intervention based on physical activity. Using a simple stepwise approach, people referring to their primary care physician for any reason were screened for their diabetes risk with a web-based app of the Findrisc questionnaire. Those at risk for T2D, i.e., with a Findrisc score >9, were invited to come back after overnight fasting to measure fasting glycaemia (FG). Those with 100 ≤ FG < 126 mg/dL were considered as people with prediabetes and compiled the Physical Activity Readiness Questionnaire (PAR-Q) to then receive a personalised prescription of physical activity. Results: Overall, 5928 people were enrolled and compiled the questionnaire. Of these, 2895 (48.8%) were at risk for T2D. Among these, FG was measured in 2168 subjects (participation rate 75%). The numbers of individuals with undetected prediabetes and T2D according to FG were 755 and 79 (34.8% and 3.6% of those assessing FG), respectively. Of the 755 subjects in the prediabetes range, 739 compiled the PAR-Q and started a personalised program of physical activity (participation rate 97%). Physicians involved in the study reported a mean of 6 min to perform the screening. Conclusions: Overall, these data suggest the feasibility of a national diabetes prevention program developed by general practitioners using a simple stepwise approach starting from a web app to intercept individuals with prediabetes.

## 1. Introduction

Type 2 diabetes (T2D) is a key risk factor for the development of cardiovascular diseases and mortality [1,2]. The incidence of T2D has constantly increased during the last decades in most countries, paralleling the trend observed with obesity [3]. Before the appearance of frank disease, subjects at risk of T2D usually develop prediabetes, a condition where blood sugar levels are higher than normal but not high enough to diagnose T2D [4]. Individuals in the prediabetes range have a fasting glucose (FG) between 100 and 126 mg/dL, and/or an HbA1c ranging from 5.7% to 6.4%, and/or a glucose level between 140 and 199 mg/dL after 2 h from a 75 g oral glucose tolerance test (OGTT) [5]. Prediabetes can be targeted to halt the development of T2D or even to fully reverse this pathological status. Indeed, the risk of progression from prediabetes to frank T2D is reduced by lifestyle interventions based on physical activity, as demonstrated by the Diabetes Prevention Program and other large trials [6,7,8]. However, it is unclear if such an approach is feasible when applied on a nationwide scale.

Previous attempts of screening on a large scale provided mixed results in terms of patients’ participation, feasibility, and efficacy in terms of detection of prediabetes or T2D [9,10,11,12,13]. Broadly, active seeking of possible cases of T2D by primary care physicians yielded a much higher participation or screening rate compared with mail-distributed, self-administered risk scores [11,12,13]. On the other hand, procedures in general practice should not be time-consuming to increase the likelihood of success of a prevention program. 

To explore the feasibility of a T2D prevention program on a national scale based on a simple stepwise approach, we involved multiple associations of general practitioners located in ten different regions in Italy with the goal of enrolling 6000 random subjects without known metabolic diseases to detect individuals with prediabetes and prescribe them an intervention based on physical activity. Here, we report the results of the screening phase.

## 2. Methods

### 2.1. Study Design

This study aims to explore the feasibility of a large diabetes prevention program. Thus, it is a cross-sectional screening study without a prospective collection of outcomes. Using a simple stepwise approach, people referring to their primary care physician for any reason were screened for their diabetes risk with a web-based app of the updated Findrisc questionnaire [7,14]. Briefly, the Findrisc utilises a scoring system that evaluates various risk factors, including age, physical activity, family history of diabetes, body mass index, waist circumference, previous history of hyperglycemia, use of anti-hypertensive drugs, and consumption of fruits and vegetables. This structured assessment assigns a specific score to each item, and the overall score is derived by summing up the scores from all items, resulting in a range from 0 to 26 [7]. Those with a score >9 were invited to come back after overnight fasting to measure fasting glycaemia (FG). The combination of a cut-off of nine plus the measurement of FG was selected based on a previous cost-effectiveness analysis [15]. Capillary FG was measured to minimise the duration of the procedure and allow its execution in the general practitioner’s office. Previous data support a good correlation between capillary and venous blood glucose, suggesting the possibility of using capillary FG for screening purposes [16]. 

**Figure 1:**
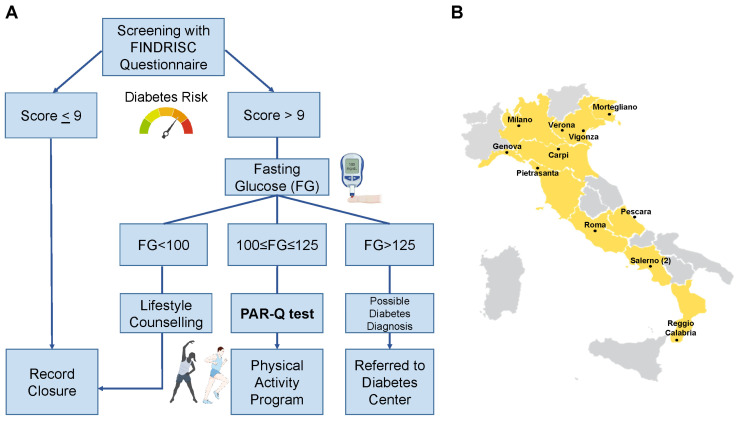
**Design of the study.** Flow for the enrolled individuals (**A**) and locations of the general practitioners’ associations involved throughout Italy (**B**).

Using the criteria of the American Diabetes Association [17], those with FG < 100 mg/dL were considered not at risk and received general counselling on healthy lifestyles; those with FG ≥ 126 mg/dL were considered as possible cases of T2D and were referred to the Diabetes Center for further examination, and those with 100 ≤ FG < 126 mg/dL were considered as people with prediabetes. This population was invited to compile the Physical Activity Readiness Questionnaire (PAR-Q) [18]. According to results, people were categorised as (i) low risk, i.e., individuals who are asymptomatic and had no more than one risk factor; (ii) moderate risk, i.e., subjects older than 45 years old with two or more risk factors; (iii) high risk, i.e., individuals with known cardiovascular or pulmonary diseases. These individuals then received a personalised prescription for physical activity. An example of tailored prescription, based on existing guidelines or on solid evidence [19,20,21,22,23,24,25], for each category is reported in Supplementary Table S1. The design of the study is summarised in Figure 1A.

### 2.2. Web-Based Application and Survey 

The dedicated web-based application was sequential and started with an Italian-translated version of the Findrisc questionnaire [14], previously validated [15]. The program asked for the measurement of FG only for individuals with a score >9. In the same manner, the app asked to compile the Italian-translated version of the PAR-Q only for subjects with FG in the prediabetes range. Screenshots from the application are reported in Supplementary Figure S1.

A survey was conducted among physicians to ask 1—the mean time spent compiling the questionnaire, 2—if they were satisfied with the initiative, and 3—if they were satisfied with the web app. Only binary responses (yes/no) were allowed for the latter two questions.

### 2.3. Statistical Analysis 

The main goal of the project was to assess the feasibility of a nationwide diabetes prevention program. Thus, there was no pre-specified primary outcome. Endpoints of interest were the participation rate of screened subjects, the time spent by physicians to perform the screening, the satisfaction of the physicians, and the baseline characteristics, i.e., those requested by the Findrisc, of the population enrolled according to their degree of risk. To summarise such descriptive statistics, subjects were categorised as low risk (Findrisc score < 10), moderate risk (10 ≤ score ≤ 15), high-risk (16 ≤ score ≤ 20), and very high risk (score > 20), adapting previously reported strata according to the selected cut-off [7,15]. Among those subjected to FG measurement, the same variables were also compared in individuals with vs without any form of dysglycemia. The distribution of variables was assessed using the Shapiro-Wilk test. Continuous variables were compared among groups with Mann-Whitney U or the Kruskal-Wallis tests. For categorical variables, we used Fisher’s exact or the Chi-squared test. *p* values are reported in the relative tables. We used GraphPad Prism version 10.1.0. for calculations and Microsoft Excel 2016 for graph preparation. Parts of the figures were drawn by using pictures from Servier Medical Art. Servier Medical Art by Servier is licensed under a Creative Commons Attribution 3.0 Unported License (https://creativecommons.org/licenses/by/3.0/).

## 3. Results

Primary care physicians from 12 different associations were involved in the study, covering a total of 10 Italian regions: 5 in the north, 3 in the centre, and 2 in the south of Italy. The location of the associations involved is presented in Figure 1B.

**Figure 2:**
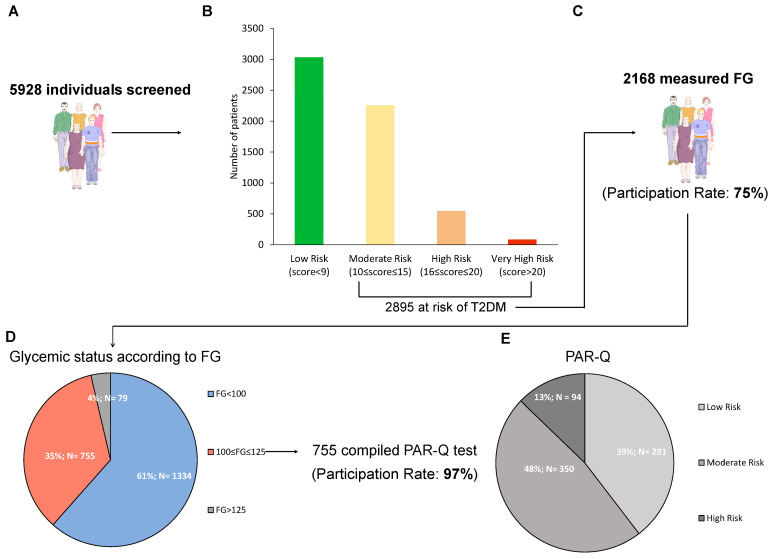
Results of the screening. Number of individuals enrolled in study (**A**), number of patients in each diabetes risk category according to Findrisc (**B**), number of subjects measuring capillary fasting glucose (FG) with the relative participation rate (**C**), number and percentage of individuals in each stratum of glycemic status according to capillary FG (**D**), and participation rate to the program of physical activity, along with number and percentage of individuals in each risk category according to PAR-Q (**E**). Low risk: individuals with no more than one risk factor; Moderate risk: subjects older than 45 years old with ≥2 risk factors; High risk: individuals with known cardiovascular or pulmonary diseases.

At the end of the study, 5928 people were screened with the Findrisc questionnaire (Figure 2A). Of these, 2895 (48.8%) had a score >9 and were thus at risk for T2D. In detail, 3033 (51.2%) of subjects were at low risk, 2258 (38.1%) at moderate risk, 547 (9.2%) at high risk, and 90 (1.5%) at very high risk (Figure 2B). All the variables of the Findrisc had the expected increasing prevalence among these four groups, with the exception of age (Table 1). Indeed, individuals in the group with the highest risk score, compared with the other three groups, were more often males, had higher mean body mass index (BMI), had more often a previous episode of hyperglycemia, made larger use of antihypertensive drugs, had more often familiarity for T2D, had more often larger waist circumferences, while they were less often daily consumers of fruit and vegetables and practised less often daily physical activity. 

Among the 2895 at risk, FG was measured in 2168 subjects (participation rate 75%) (Figure 2C). The numbers of individuals with undetected prediabetes and T2D according to FG were 755 and 79 (34.8% and 3.6% of those assessing FG), respectively (Figure 2D). When comparing the groups of subjects with (*n* = 834) vs those without (*n* = 1334) any form of dysglycemia, people in the first group had higher values of weight, waist circumference, and BMI, had more often previously faced an episode of reported hyperglycemia and were more often users of antihypertensive drugs (Table 2). 

Of the 755 subjects in the prediabetes range, 735 compiled the PAR-Q (participation rate 97%). When stratifying according to PAR-Q, 291 (39.6%) of the subjects were at low risk, 350 (47.6%) at moderate risk, 96 (12.8%) at high risk (Figure 2E), and all received a tailored prescription of physical activity (examples provided in Supplementary Table S1). 

A survey conducted among the physicians involved in the study evidenced a mean time to compile the Findrisc of 5.7 ± 2.1 min, with a high rate of satisfaction with the initiative (68%) but a low rate of satisfaction with the web application (34%). 

## 4. Discussion

Structured interventions on lifestyles and, in particular, physical activity are able to curb the progression of prediabetes to frank T2D [6]. However, the feasibility of developing such an intervention at the population level on a national scale is uncertain. In this pilot study, we involved multiple associations of primary care physicians located on the Italian territory to screen people referring to their general practitioners with a web-based app of the Findrisc questionnaire. The results obtained support the feasibility of this simple stepwise approach based on the compilation of such a questionnaire followed by the measurement of capillary FG in subjects at risk. Indeed, the mean time spent by the physicians involved was reasonable, and the program has been developed as planned, notwithstanding the struggle ascribable to the COVID-19 pandemic, which influenced and still affects the normal medical routine [25].

The physicians involved reported a good degree of satisfaction with the overall initiative but not with the application. This is possible because a dedicated web app was developed ad hoc for the study, but such a program did not interact with the electronic health record commonly used in routine care, an aspect that slightly slowed the procedure. This aspect might be implemented by future similar studies.

Relatively to the approach used for the screening, we might have missed some patients with prediabetes or even with frank T2D. Indeed, it is conceivable that selected individuals do have altered glycemic values when exposed to OGTT despite a normal FG range. However, the primary goal of this study was to explore the feasibility of a prevention program conducted on a national scale and not to establish the best method to detect diabetes or prediabetes. Thus, the approach selected was the one with the best combination of effectiveness and ease of development, according to previous literature [15]. In addition, the observation that a recent screening program developed through gazebos organised during public initiatives and verifying the diagnosis yielded a comparable incidence of prediabetes, i.e., roughly half of those at risk, according to Findrisc [10], reassures about the ability of our program to properly identify such population. 

When developing preventive programs on a large scale, a key aspect is the participation rate of the individuals screened. Previous attempts at diabetes screening the population by sending the questionnaire through mail yielded very low participation rates, e.g., 18% [12]. On the contrary, programs actively involving primary care physicians obtained a very high participation rate [11]. Other recent studies also explored different approaches. For instance, one study was conducted with the use of extension agents enrolling random people at the health fairs or at the library and community events, testing a risk score similar to Findrisc coupled with HbA1c measurement through point-of-care [26]. However, participation rates were generally low [26]. Other studies took advantage of general practitioners to identify patients at risk but then proposed an eHealth-based intervention to perform the lifestyle intervention [27]. Even in this case, the participation rate was low even though the intervention was effective in promoting weight loss in those adhering to the program [27]. Another issue linked to eHealth interventions is that racially and ethnically diverse populations with limited levels of educational attainment might not be reached by such programs [27]. Other approaches explored a program of diabetes screening through community pharmacies [28,29]. In this case, the number of cases detected was low, and the relative costs were high, especially because a large number of individuals at risk did not refer the condition to their general practitioner [28]. Another issue associated with such an approach is that a low number of pharmacies might adhere to the initiative, thus not covering a large portion of the target population [30]. Our results substantiate the observation that general practitioners play a key role when developing such programs. More broadly, they support the notion that patients are more willing to undertake screening if they are approached whilst accessing healthcare for a different purpose [28]. 

Data from a previous clinical trial exploring the effect of large-scale screening for diabetes on the incidence of subsequent cardiovascular or diabetes-related mortality showed no benefit of screening *per se* on such hard endpoints within the following 10 years [9]. This lack of benefit might be ascribed to a number of reasons, including the inability of programs to detect the disease in all subjects and, eventually, the inadequate response implemented by physicians and patients after the diagnosis. To this respect, another study showed that a subgroup of this population treated intensively for multiple cardiovascular risk factors obtained a small, albeit not significant, benefit in terms of reduced incidence of cardiovascular diseases and mortality [31,32]. Follow-up duration in most of these trials may have been too short to detect an effect on health outcomes [33]. However, a limited benefit on hard endpoints has also been observed in a long-term follow-up of the Diabetes Prevention Program, despite the high efficacy of lifestyle modification in reducing the rate of progression of prediabetes to T2D [34]. These findings are counterintuitive, considering that early intervention targeting glycemic control is associated with a long-term benefit [35,36]. Possible explanations, among others, include a lack of effect of different treatments on the pathogenic mechanisms of the disease and the possibility that an even earlier interception of the disease trajectory might be needed to limit the noxious effect of glucose imbalances. Whatever the case, diabetes prevention should remain a major goal of public health in order to decrease the burden of multiple complications and the relative costs. This postulate has been recently endorsed also by the US Preventive Services Task Force, which recommends screening for prediabetes and type 2 diabetes in adults aged 35 to 70 years, especially those who are overweight or obese. Clinicians should offer or refer patients with prediabetes to effective preventive interventions (B recommendation) [33].

Our study has several limitations. Given its explorative nature assessing the feasibility, and not the efficacy, of a nationwide program, we did not compare the effectiveness of different screening approaches in detecting prediabetes or T2D. For the same reason, we did not include a control group, e.g., with no intervention prescribed, in those with detected prediabetes. Thus, we are not able to establish if such a wide program has the same efficacy in reducing the incidence of T2D of previously published studies enrolling selected patients with prediabetes [6]. In addition, we did not measure the adherence of individuals to the prescribed physical activity programs, nor their satisfaction and their quality of life. Such key aspects, along with the efficacy of the intervention on the subsequent incidence of T2D, warrant further investigation in future prospective studies. 

In summary, here we show that a nationwide diabetes prevention program is feasible. A simple stepwise approach developed by primary care physicians using the Findrisc questionnaire followed by the measurement of FG allowed the identification of a large number of individuals with prediabetes, most of whom initiated a program of physical activity. Additional studies are needed to establish the usefulness and efficacy of such an approach in limiting prediabetes progression on a large scale. 

## Figures and Tables

**Table 1:** Summary of the variables included in the Findrisc questionnaire in the four categories of risk.

Variable		Low Risk (Score < 9)	Moderate Risk (10 ≤ score ≤ 15)	High Risk (16 ≤ Score ≤ 20)	Very High Risk (Score > 20)	*p*-Value	Overall
**Age (years)**		55 (48–61)	55 (48–61)	55 (49–61)	55.5 (49–61)	0.885	55 (48–61)
**Male, n (%)**		1395 (46%)	969 (43%)	227 (42%)	52 (52%)	<0.005	2643 (45%)
BMI (Kg/m^2^) *		23.4 (21.4–25.4)	26.37 (24.2–29.0)	29.41 (26.6–32.5)	31.8 (30.1–33.9)	<0.0001	24.98 (22.6–27.7)
**Weight (kg)**		67 (58–76)	75 (65–85)	82 (72–94)	90 (82–98)	<0.0001	71 (62–81)
**Consumption of fruit and vegetables**	Everyday	2516 (83%)	1600 (70.9%)	367 (67.1%)	45 (50%)	<0.0001	4528 (76.4%)
	Not everyday	517 (17%)	658 (29.1%)	180 (32.9%)	45 (50%)		1400 (23.6%)
**Known hyperglycemia**	No	3004 (99%)	2094 (92.7%)	330 (60.3%)	2 (2.2%)	<0.0001	5430 (91.6%)
	Yes	29 (1%)	164 (7.3%)	217 (39.7%)	88 (97.8%)		498 (8.4%)
**Exercise for at least 30 min almost every day**	Yes	1882 (62.1%)	952 (42.2%)	148 (27.1%)	15 (16.7%)	<0.0001	2997 (50.6%)
	No	1151 (37.9%)	658 (57.8%)	399 (72.9%)	75 (83.3%)		2931 (49.4%)
**Antihypertensive drugs**	No	2745 (90.5%)	1490 (66%)	199 (36.4%)	19 (21.1%)	<0.0001	4453 (75.1%)
	Yes	288 (9.5%)	768 (34%)	348 (63.6%)	71 (78.9%)		1475 (24.9%)
**Cases of diabetes in the family**	No	2053 (67.7%)	777 (34.4%)	57 (10.4%)	1 (1.1%)	<0.0001	2888 (48.7%)
	Yes: grandparents, uncles, cousins	655 (21.6%)	564 (25%)	119 (21.8%)	17 (18.9%)		1355 (22.9%)
	Yes: parents, brothers, sisters, children	325 (10.7%)	917 (40.6%)	371 (67.8%)	72 (80%)		1685 (28.4%)
**Waist circumference**	<94 for men; <80 for women	1653 (54.5%)	219 (9.7%)	7 (1.3%)	0 (0%)	<0.0001	1897 (31.7%)
	94 ≤ men ≤ 102; 81 ≤ women ≤ 88	910 (30%)	767 (34%)	99 (18.1%)	3 (3.3%)		1779 (30%)
	>102 for men; >88 for women	470 (15.5%)	1272 (56.3%)	441 (80.6%)	87 (96.7%)		2270 (38.3%)

* BMI = Body mass index. Data are presented as median (Q1–Q3) for continuous variables and as number (percentage) for categorical variables. +*p* values derive from Kruskal-Wallis test for continuous variables and from Chi squared test for categorical variables.

**Table 2:** Summary of the variables included in the Findrisc questionnaire in the groups of patients with (*n* = 834) or without (*N* = 1334) any form of dysglycemia according to fasting glucose.

Variable		Normoglycemic Patients (FG < 100 mg/dL)	Pre-Diabetic and Diabetic Patients (FG ≥ 100 mg/dL)	*p*-Value
**Age (years)**		55 (49–62)	55 (48–61)	0.289
**Male, n (%)**		560 (42%)	388 (47%)	0.041
BMI (Kg/m^2^) *		26.6 (24.5–29.4)	27.9 (25.4–31.2)	<0.0001
**Weight (kg)**		75 (66–85)	80 (70–91)	<0.0001
**Consumption of fruit and vegetables**	Everyday	902 (67.6%)	571 (68.5%)	0.705
	Not everyday	432 (32.4%)	263 (31.5%)	
**Known hyperglycemia**	No	1186 (89%)	620 (74%)	<0.0001
	Yes	148 (11%)	214 (26%)	
**Exercise for at least 30 min almost every day**	Yes	487 (36.5%)	314 (37.6%)	0.615
	No	847 (63.5%)	520 (62.4%)	
**Antihypertensive drugs**	No	845 (63.3%)	432 (51.8%)	<0.0001
	Yes	489 (36.7%)	402 (48.2%)	
**Cases of diabetes in the family**	No	382 (28.6%)	250 (30%)	0.022
	Yes: grandparents, uncles, cousins	364 (27.3%)	184 (22.1%)	
	Yes: parents, brothers, sisters, children	588 (44.1%)	400 (47.9%)	
**Waist circumference**	<94 for men; <80 for women	126 (9.4%)	46 (5.5%)	<0.0001
	94 ≤ men ≤ 102; 81 ≤ women ≤ 88	412 (30.9%)	221 (26.5%)	
	>102 for men; >88 for women	796 (59.7%)	567 (68%)	

* BMI = Body mass index. Data are presented as median (Q1–Q3) for continuous variables and as number (percentage) for categorical variables. +*p* values derive from the Mann-Whitney U test for continuous variables and from the Fisher exact test for categorical variables.

## Data Availability

The identified dataset can be requested to francesco.prattichizzo@multimedica.it.

## Supplementary Materials

The following supporting information can be downloaded at: https://www.mdpi.com/article/10.3390/jcm13041127/s1, Figure S1: Screenshots from the web-based app used by the general practitioners showing the used version of the Findrisc and PAR-Q questionnaires; Table S1: Examples of physical activity prescriptions tailored according to category of risk.
