# Supplementary material for: Feasibility of a Type 2 Diabetes Prevention Program at Nationwide Level in General Practice: A Pilot Study in Italy
Source: J Clin Med. 2024 Feb 16;13(4):1127. doi: 10.3390/jcm13041127 (PMC10888610; doi:10.3390/jcm13041127)

Supplementary Material

Supplement to: La Grotta R., et al. Feasibility of a diabetes prevention program at nationwide level in general practice: a pilot study in Italy.

Supplementary Table S1. Examples of physical activity prescriptions tailored according to category of risk. Source references for evidence-based prescriptions are provided.

Category of risk (PAR-Q)	Prescription	Resistance Training	Example	Reference
**Low Risk (Younger individuals, asymptomatic, meeting no more than one risk factor threshold)**	Recommend moderate-intensity aerobic activities	150 minutes per week. Suggest incorporating two or more days a week focusing on major muscle groups using body weight exercises or weights	Encourage activities such as brisk walking, cycling, swimming, or dancing for 30 minutes on most days of the week	1,2
**Moderate Risk (Older individuals over 45 years old with two or more risk factors)**	Advocate a combination of moderate to vigorous aerobic exercises	Emphasize incorporating resistance exercises targeting major muscle groups using weights or resistance bands at least two days a week	Recommend alternating between jogging or cycling for 25-30 minutes on most days along with high-intensity interval training (HIIT) twice a week	3,4
**High Risk (Individuals with known cardiovascular or pulmonary diseases)**	Advocate supervised exercise programs focusing on low to moderate intensity, gradually progressing over time.	Highlight the importance of focusing on cardiac rehabilitation exercises under professional guidance to ensure safety and proper monitoring	Initiate with supervised low-impact exercises like water aerobics or stationary cycling for 20-30 minutes, three times a week, gradually increasing duration and intensity under supervision	5,6

References:

1. Haskell, W. L., et al. (2007). Physical activity and public health: updated recommendation for adults from the American College of Sports Medicine and the American Heart Association. Medicine and Science in Sports and Exercise, 39(8), 1423-1434. DOI: 10.1249/mss.0b013e3180616b27.
2. Garber, C. E., et al. (2011). Quantity and quality of exercise for developing and maintaining cardiorespiratory, musculoskeletal, and neuromotor fitness in apparently healthy adults: guidance for prescribing exercise. Medicine and Science in Sports and Exercise, 43(7), 1334-1359. DOI: 10.1249/MSS.0b013e318213fefb.
3. Weston, K. S., et al. (2014). High-intensity interval training in patients with lifestyle-induced cardiometabolic disease: a systematic review and meta-analysis. British Journal of Sports Medicine, 48(16), 1227-1234. DOI: 10.1136/bjsports-2013-092576.
4. American College of Sports Medicine. (2009). American College of Sports Medicine position stand. Progression models in resistance training for healthy adults. Medicine and Science in Sports and Exercise, 41(3), 687-708. DOI: 10.1249/MSS.0b013e3181915670.
5. Taylor, R. S., et al. (2014). Exercise-based rehabilitation for heart failure. Cochrane Database of Systematic Reviews, 4(4), CD003331. DOI: 10.1002/14651858.CD003331.pub4.
6. Fletcher, G. F., et al. (2013). Exercise standards for testing and training: a scientific statement from the American Heart Association. Circulation, 128(8), 873-934. DOI: 10.1161/CIR.0b013e31829b5b44.

Supplementary Figure S1. Screenshots from the web-based app used by the general practitioners showing the used version of the Findrisc and PAR-Q questionnaires.

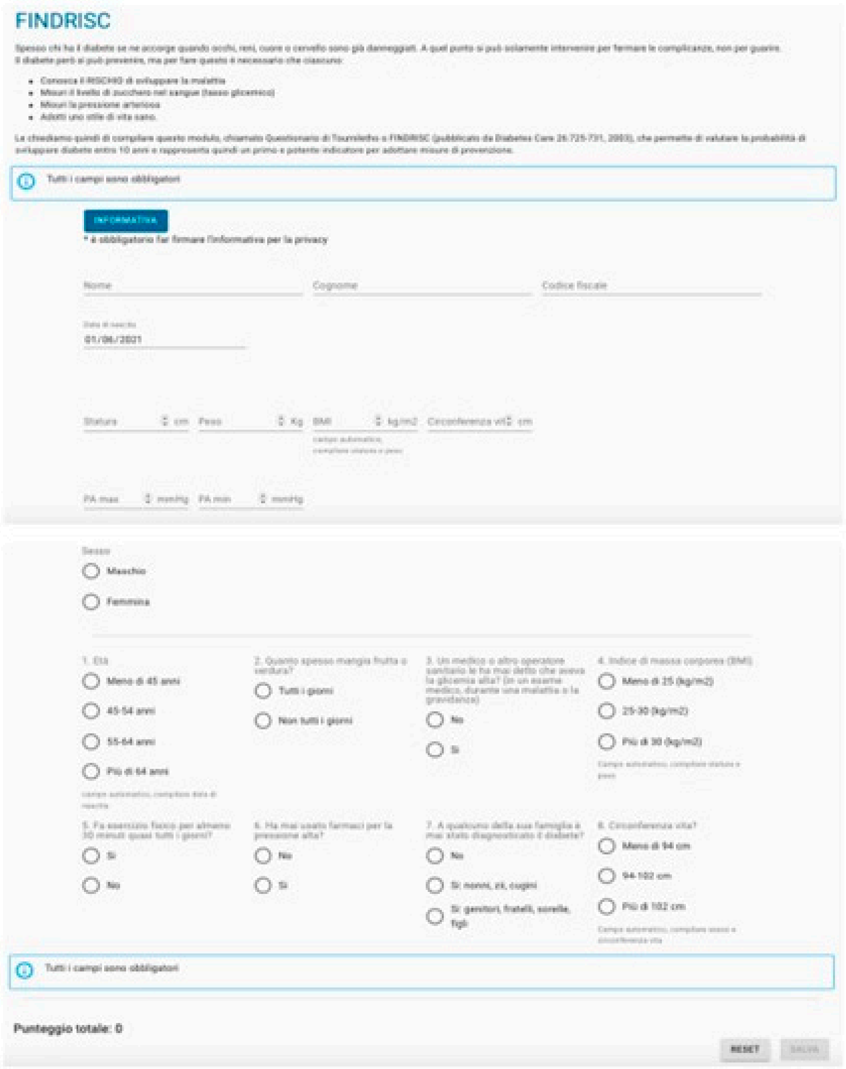

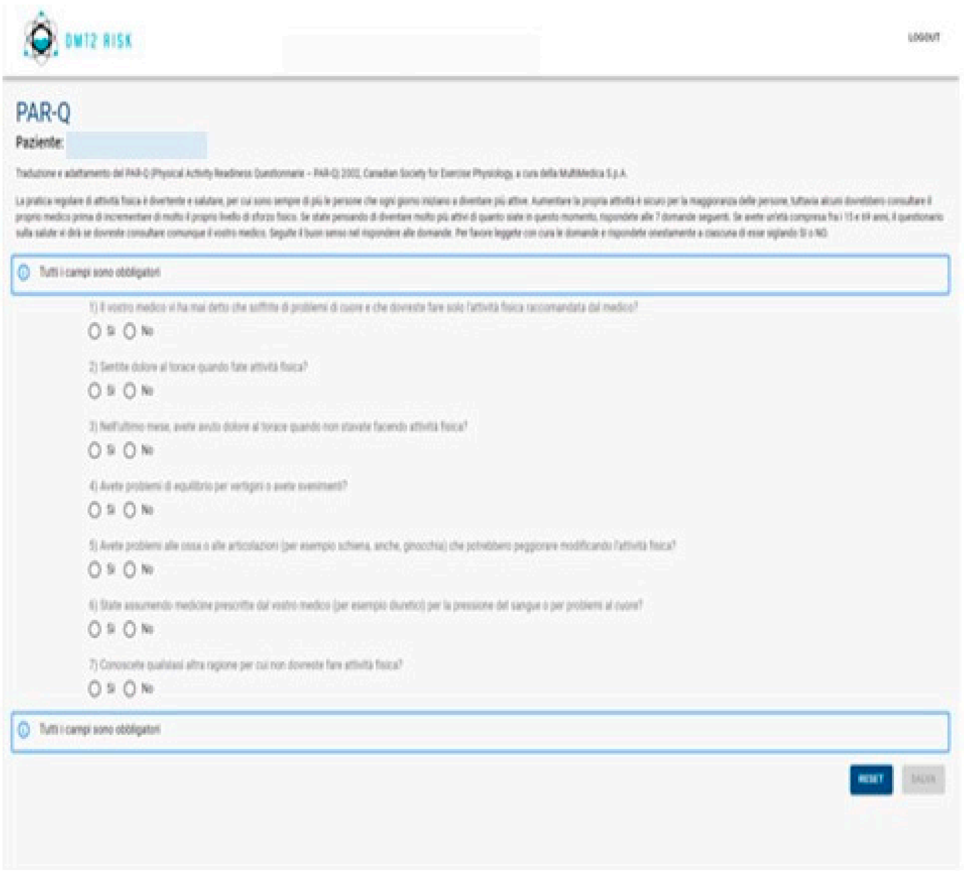

Supplement: Supplementary file 1 [file jcm-13-01127-s001.zip › jcm-2852078-supplementary.pdf]
